# Optical logic operation via plasmon-exciton interconversion in 2D semiconductors

**DOI:** 10.1038/s41598-019-45204-0

**Published:** 2019-06-24

**Authors:** Jung Ho Kim, Jubok Lee, Hyun Kim, Seok Joon Yun, Jeongyong Kim, Hyun Seok Lee, Young Hee Lee

**Affiliations:** 1 Center for Integrated Nanostructure Physics (CINAP), Institute for Basic Science (IBS), Sungkyunkwan University, Suwon, 16419 Republic of Korea; 2 Department of Energy Science, Sungkyunkwan University, Suwon, 16419 Republic of Korea; 3 Department of Physics, Chungbuk National University, Cheongju, 28644 Republic of Korea

**Keywords:** Two-dimensional materials, Nanophotonics and plasmonics, Nanowires

## Abstract

Nanophotonic devices manipulating light for high-speed computing are a counterpart of speed-limited electronic circuits. Although plasmonic circuits are a promising platform for subwavelength miniaturization, the logic-operation principle is still limited to mimicking those of photonic waveguides using phase shifts, polarization, interference, and resonance. Meanwhile, reconfigurable interconversion between exciton and plasmon engender emerging applications like exciton transistors and multiplexers, exciton amplifiers, chiral valleytronics, and nonlinear excitonics. Here, we propose optical logic principles realized by exciton-plasmon interconversion in Ag-nanowires (NW) overlapped on transition metal dichalcogenides (TMDs) monolayers. Excitons generated from TMDs couple to the Ag-NW plasmons, eventually collected as output signals at the Ag-NW end. Using two lasers, we demonstrate AND gate by modulating single excitons in Ag-NW on MoS_2_ and a half-adder by modulating dual excitons in lateral WSe_2_ and WS_2_. Moreover, a 4-to-2 binary encoder is realized in partially overlapped MoSe_2_ and MoS_2_ using four-terminal laser inputs. Our results represent great advances in communication processing for optical photonics integrable with subwavelength architectures.

## Introduction

A logic device is one of the essential platform for advanced information processing and thus the versatile logic architectures have well been developed for state-of-the-art electronics. For high speed and scalable information technology, the nanophotonics has been proposed as one of the strong counterparts to overcome the inherent limitation of electronic integrated circuits. In this reason, plasmonic logic gates also have been investigated, because plasmonic integrated circuits, which can manipulate optical information at nanoscales, possess both advantages of photonics and electronics in terms of operation speed and scalability^1–5^. However, a proper concept which utilizes the uniqueness of metallic nano-waveguides has rarely been developed. Although most researches are based on the principles to mimic photonic waveguides including phase shifts and interferences, massive and complicated device architectures, such as precisely engineered ring resonators and waveguide branches, alleviates the advantages of plasmonics at nanoscales^6–8^. Since plasmonic logic devices have been created that mimic photonic waveguides as well as electrical circuits operating at optical frequencies, therefore, a development of unique operation principle for plasmonic logics is strongly required for realizing ultra-miniaturized nanophotonic logics.

Meanwhile, hybridization methods of metallic NWs with excitonic quantum emitters, such as quantum dots and two-dimensional (2D) semiconductors have been widely investigated to modulate light signals in plasmonic waveguides^9–16^. Recently emerging 2D semiconductors of monolayer TMDs have unique merits with a vast class of materials library^17^ as well as stable exciton formation at room temperature^18^ and robust chip-integration^19^. Furthermore, Ag-NWs hybridized with TMDs enable reconfigurable interconversion of exciton-to-plasmon and/or plasmon-to-exciton for optical communications^9,10^. Herein, we propose optical logic principles using plasmon-exciton interconversions in several architectures constructed by overlapping Ag-NWs on monolayer TMDs. For modulating exciton signals, two external lasers are utilized for simplified device structures.

## Results and Discussions

**Figure 1:**
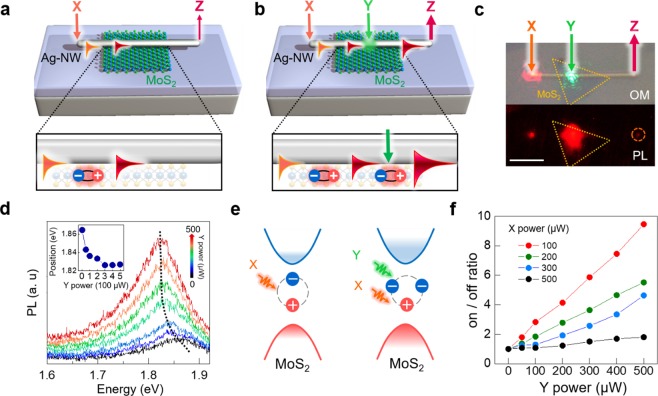
Optical modulation of exciton-plasmon interconversions. (**a,b**) Schematic illustration of the working principle of the Ag-NW/MoS_2_ switching device. (**c**) OM and PL image of the device under laser X and Y illumination. Laser X (orange arrow, 633 nm wavelength) is irradiated at the left end of Ag-NW and laser Y (green arrow, 514 nm wavelength) is irradiated on Ag-NW and MoS_2_ overlapped region. PL spectrum is detected at the right end of Ag-NW. (orange dotted line, bottom) Scale bar in the optical images of indicates 5 µm. (**d**) The collected PL spectrum variation as a function of laser Y power. When laser Y power increase, the resulting PL intensity not only increase but also move to a lower energy regime (red shift, inset) owing to trion formation at high laser power. (**e**) Illustration of trion formation under additional Y laser illumination. (**f**) On/off ratio plotted by laser Y power variation. On: X and Y lasers “on” states. Off: X laser “on” state but Y laser “off” state. On/off ratio increases as laser Y power increase, since more excitons are created. Furthermore, smaller laser X power shows a larger on/off ratio. This originates from a smaller off-state level when small laser X power is applied.

A basic operation principle and optical switching mechanism using exciton-plasmon interconversions are presented in Fig. 1. Two focused lasers named X (orange arrow) and Y (green arrow) are illuminated on Ag-NW overlapped on monolayer MoS_2_ (see Methods). When the laser X is illuminated at the Ag-NW left-end, the laser is coupled to surface plasmon polaritons (SPPs) propagating along the Ag-NW, exciting excitons in the MoS_2_ layer. The excitons in MoS_2_ sequentially couple to SPPs due to Förster resonance energy transfer^20^ and propagate along the NW, reaching the Ag-NW right-end (Z) to emit SPP signals via light scattering (Fig. 1a)^9^. The SPP signals collected by photoluminescence (PL) at Z are modulated by the laser Y as presented in Fig. 1b. The green laser spot at the MoS_2_ region (Y) generates the exciton which couples to SPPs in the Ag-NW. Notably, when the green laser is irradiated in the middle part of Ag-NW, the laser light cannot be coupled to SPP, because the momentum compensation for longitudinal direction between SPP and incident light is not available at the midsection of NW. The laser only excites MoS_2_ exciton and the exciton is coupled to SPP via Förster resonance energy transfer^20^. Both laser irradiations at the Ag-NW left-end and at the Ag-NW/MoS_2_ area (X and Y) increase the PL intensity at the Ag-NW right-end (Z). The optical micrograph (OM) and PL images are shown in Fig. 1c. In the PL image, we can observe PL emission at the Z position (orange dotted circle). To operate this device as an optical switch, we fix the laser X and modulate the laser Y power. As the laser Y power increases, the detected PL intensity subsequently increases. (Fig. 1d) If we define the PL intensity when only laser X is irradiated as “off-current”, as in electronic devices, laser Y modulation can be interpreted as “on-current” modulation. In addition, the PL intensity moves to a lower energy regime (red shift, inset) which indicates trion formation^21^. As we excite more photocarriers from addition Y laser irradiation, the excess charges couple with excitons and create trions, as illustrated in Fig. 1e. Since initial PL intensity with only laser X irradiation (“off-current”) can be manipulated by the laser X power, the resulting on/off ratio also varies. The on/off ratio reaches ~10 when the laser X power is smallest since the difference of PL intensity stands out when “off-current” is 100 μW. (Fig. 1f)

**Figure 2:**
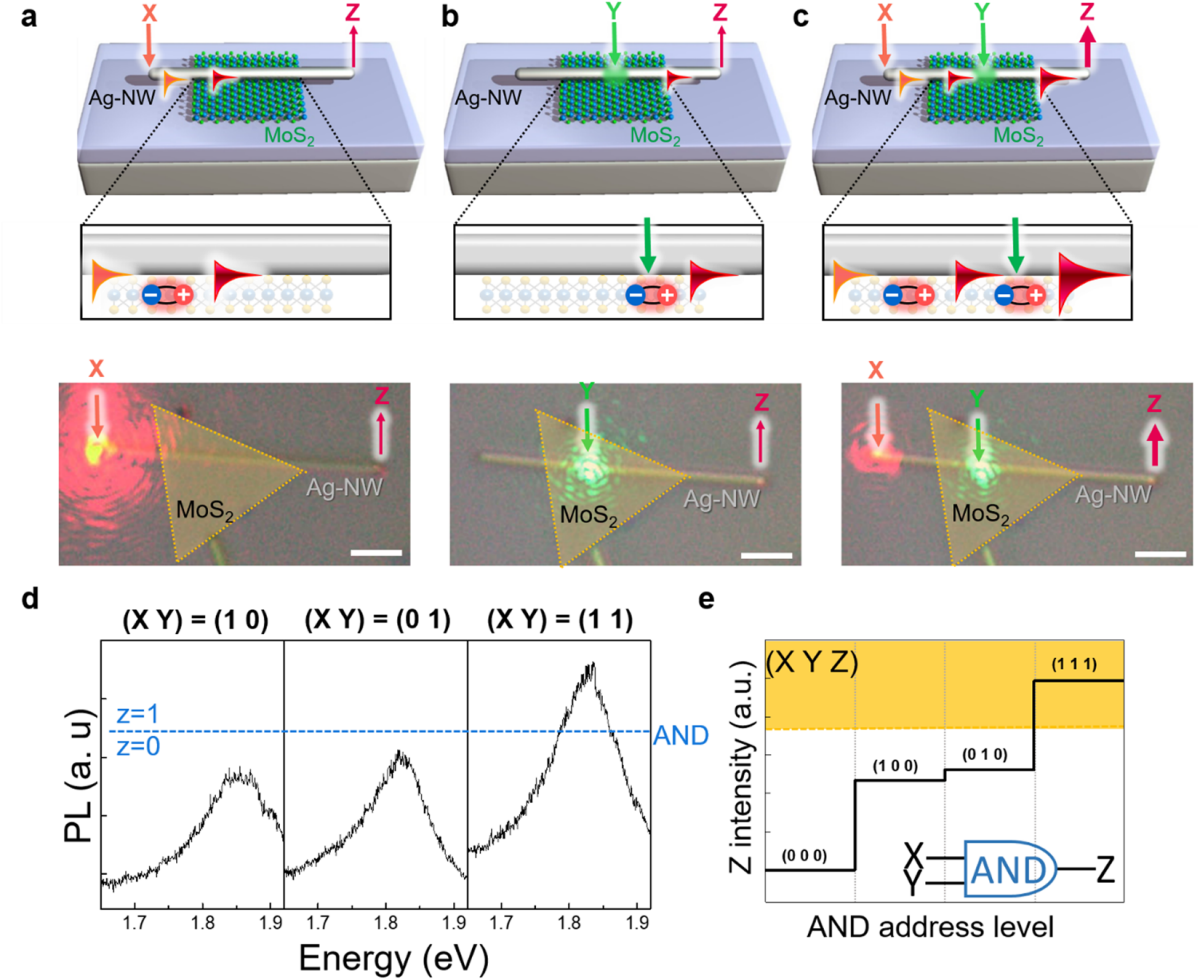
Signal modulation for AND logics. (**a**) Schematic of the laser X (orange arrow, wavelength of 633 nm) irradiation at the left end of the partially overlapped Ag-NW on MoS_2_. (**b**) Schematic of the laser Y (green arrow, wavelength of 514 nm) irradiation at the Ag-NW and MoS_2_ overlapped region. (**c**) Schematic of the laser X and laser Y irradiation. The generated signals are monitored at the Ag-NW right-end (Z). Each OM image overlaid with false-colored MoS_2_ flakes. Scale bars in the optical images of (**a-c**) indicate 5 µm. (**d**) The PL spectra at Z for the three combinations of the X and/or Y laser “on” = “1” and off” = “0” states. Blue dashed line indicate the “1” and “0” states for the AND logic gates. (**e**) The PL intensity collected at Z displays the AND logic gates with the yellow colored region depicting the “1” states.

To utilize this concept for logic operation, we first irradiate laser X (“on” state), which is detected as MoS_2_ PL at Z position. Subsequently, we irradiate laser Y, which also results as a PL spectrum at the NW end. When we irradiate both positions with laser X and Y, the following PL intensity is nearly doubled. Schematic illustration and corresponding OM images are presented in Fig. 2a–c. The combination of the described two-laser modulation allows optical logic operation. The PL intensities collected at Z for three combinations of X and/or Y laser “on” = “1” and “off” = “0” states are displayed in Fig. 2d. For (X Y) = (1 1), the maximum PL intensity at Z is approximately two times larger than that of (X Y) = (1 0) and (0 1), respectively. When the “on” state is defined as an upper level of the 2/3 intensity of the maximum PL, the AND gate of (X Y Z) = (0 0 0) (1 0 0) (0 1 0) (1 1 1) can be obtained (Fig. 2e)^2,22,23^.

**Figure 3:**
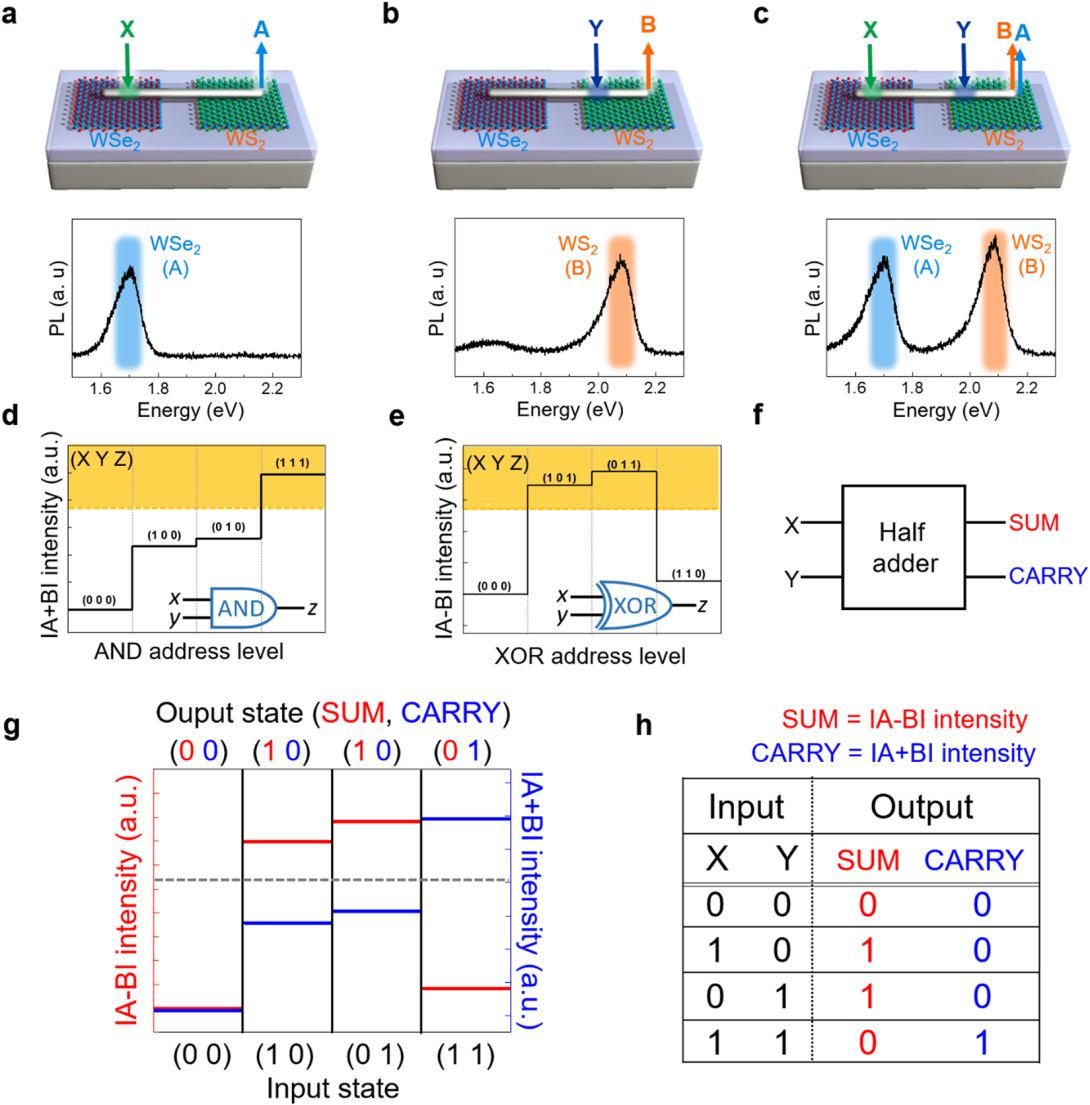
AND, XOR and half-adder operation. (**a**–**c**) Schematics of the three combinations of laser illuminations: laser X (green arrow, wavelength of 514 nm) at the Ag-NW/WSe_2_ region and laser Y (dark blue arrow, wavelength 405 nm) at the Ag-NW/WS_2_ region for the Ag-NW bridged WSe_2_/WS_2_ device (top image). The WSe_2_ (A) and WS_2_ (B) exciton spectra collected at Z (bottom image). Combinations of the $|A+B|$ and $|A-B|$ intensities collected at Z are displayed for the (**d**) AND and (**e**) XOR logic operations. The yellow region illustrates the “1” state. (**f**) The simplified half-adder diagram with the X and Y inputs and the SUM and CARRY outputs. (**g**) Optical half-adder demonstration using the laser X and Y inputs and the $|A+B|$ and $|A-B|$ intensity outputs for SUM and CARRY. The gray dotted line represents the reference of the “1” and “0” states. (**h**) A truth table of the resulting half-adder.

One of the unique features of plasmon-exciton interconversion devices is to achieve robust multiplexing of multiple exciton signals to SPPs in a single Ag-NW with a simple architecture^9^. Herein, a half-adder operation with AND and XOR logics using lateral WSe_2_ and WS_2_ layers interconnected by a Ag-NW bridge is demonstrated. Figure 3 demonstrates such a concept. By illuminating the laser X onto the Ag-NW/WSe_2_ region, the WSe_2_-exciton-coupled SPP signals are exclusively collected at the Ag-NW right-end via light scattering (bottom image, signal A), after passing through the Ag-NW/WS_2_ region. The light emission directly from the WS_2_-exciton-related signals (2.08 eV) is not allowed due to a larger bandgap than 1.7 eV of WSe_2_ (details are presented in Fig. S1). By illuminating the laser Y onto the Ag-NW/WS_2_ region, the WS_2_-exciton-coupled SPP signals are collected at the Ag-NW right-end via light emission (bottom image, signal B) as presented in Fig. 3b. Under both laser (X and Y) illuminations at the WSe_2_ and WS_2_ regions, individual SPPs coupled from the WSe_2_ and WS_2_ excitons are multiplexed in a single Ag-NW propagating along the Ag-NW and de-multiplexed at the Ag-NW right-end (Fig. 3c). The de-multiplexed PL spectra are individually presented for A and B signals separately (bottom image), where the energy difference between A and B (~0.38 eV) can be resolved by the photodetectors. Advanced logic gates are hereafter demonstrated by the simple operation of de-multiplexing the A and B signals. Figure 3d presents the $|A+B|$ and $|A-B|$ intensities measured from PL intensities at the Ag-NW right-end (Z). Although an additional computation is required for $|A+B|$ and $|A-B|$ signal processing, such a concept can be utilized for advanced logic operations such as AND and XOR gate. The “on” state is defined as an upper level of the 2/3 intensity of $|A+B|$ and $|A-B|$. The AND gate of (X Y Z) = (0 0 0) (1 0 0) (0 1 0) (1 1 1) and the XOR gate of (X Y Z) = (0 0 0) (1 0 1) (0 1 1) (1 1 0) are illustrated in Fig. 3d and e, respectively.

In electronics, conventional half-adder with two-terminal inputs (X and Y) can be achieved for SUM and CARRY output signals via a combination of AND and XOR. This operation diagram is simplified for the X and Y terminal inputs and SUM and the CARRY terminal outputs (Fig. 3f). The AND and XOR operation allows a half-adder operation. Figure 3g represents both $|A-B|$ and $|A+B|$ intensity plots as output states corresponding to each (X Y) input state, where the gray dashed line is refers to the “1” state. The output states of $|A-B|$ and $|A+B|$ correspond to the SUM (red) and CARRY (blue) states. The resulting truth table is summarized in Fig. 3h^22,24^.

**Figure 4:**
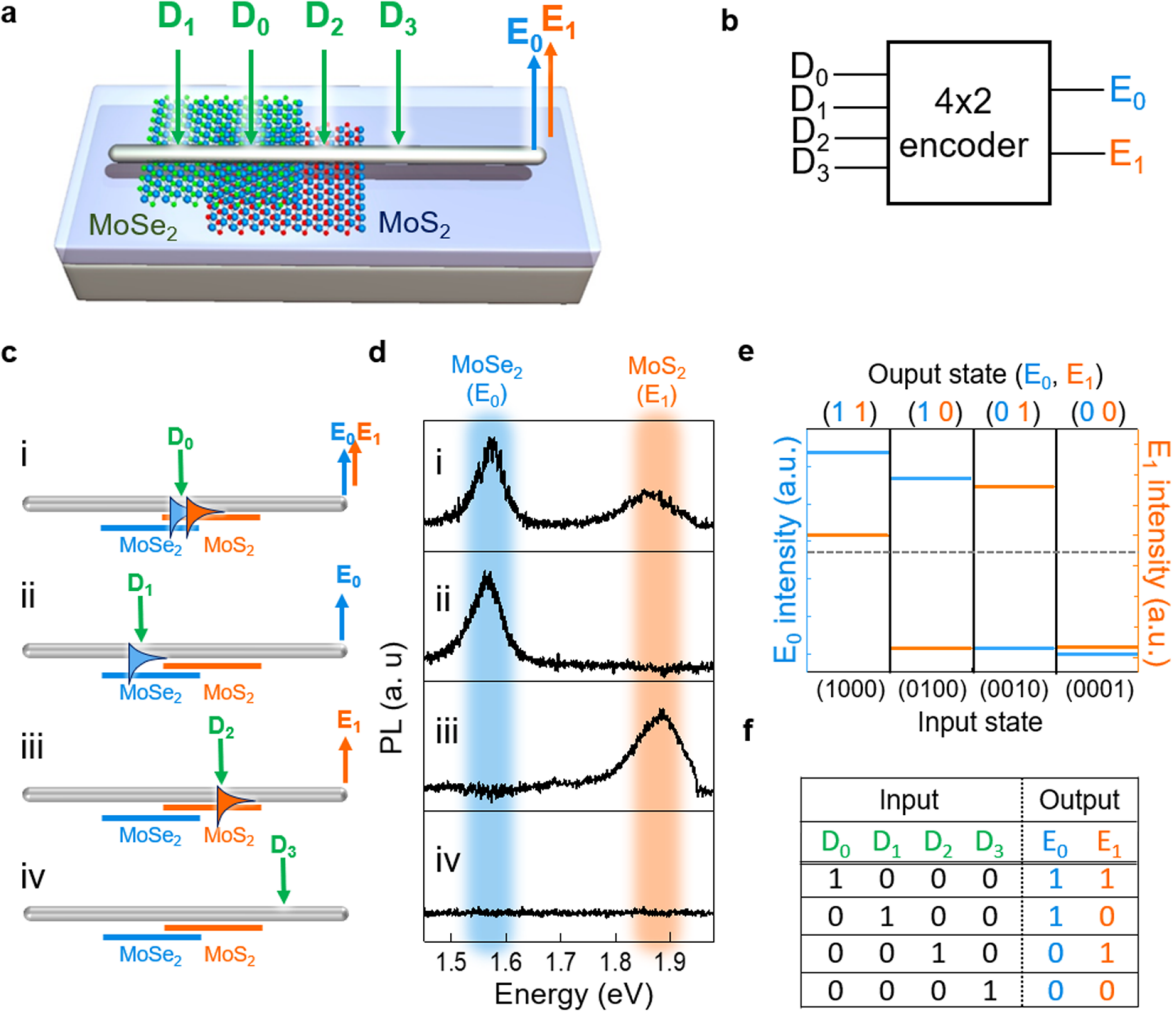
4-to-2 binary encoder operation. (**a**) Schematic of the Ag-NW on a partially stacked MoSe_2_/MoS_2_ bilayer and the laser (green arrow, wavelength of 514 nm) illuminations at four-terminals: MoSe_2_/MoS_2_ bilayer (D_0_), MoSe_2_, (D_1_), MoS_2_ (D_2_), and no TMDs (D_3_) regions. The E_0_ and E_1_ output signals collected at the Ag-NW right-end. (**b**) The simplified 4-to-2 binary encoder consisting of four input terminals (D_0_, D_1_, D_2_, and D_3_) and two output terminals (E_0_ and E_1_). (**c**) The four combinations of light illuminations at the input terminals and the corresponding MoSe_2_ (E_0_) and MoS_2_ (E_1_) exciton-related signals collected at the Ag-NW right-end (output terminal) (**d**) The collected PL spectra for corresponding combinations of light illuminations at the input terminals. (**e**) The measured E_0_ (left axis) and E_1_ (right axis) intensity plots as a function of the four different input states. The gray dashed line illustrates the reference of the “1” state for the output states and (**f**) the corresponding truth table depicting the conventional 4-to-2 binary encoder.

A versatile optical logic operation can also be obtained by constructing the van der Waals heterostructures in 2D semiconductors. An example of a 4-to-2 binary encoder is demonstrated by implementing heterostacked MoSe_2_/MoS_2_ layers. Figure 4a presents a schematic of the operation concept of 4-to-2 binary encoders in a Ag-NW partially overlapped on MoSe_2_/MoS_2_ heterobilayers. Under the input laser illumination at four different positions (D_0_, D_1_, D_2_, and D_3_ input terminals), modulated and multiplexed excitons-coupled SPPs are collected as two-type binary signals (E_0_ and E_1_) via light scattering at the Ag-NW right-end. The simplified diagram of the 4-to-2 binary encoder with four-terminal inputs and two-terminal outputs is presented in Fig. 4b. Figure 4c,d illustrate the proposed operation principles and results. By illuminating the laser at the Ag-NW overlapped on MoSe_2_/MoS_2_ heterobilayer region (D_0_ = 1), both MoSe_2_ (E_0_) and MoS_2_ (E_1_) -excitons are coupled to the SPPs and both E_0_- and E_1_-related PL spectra are collected at the Ag-NW right-end (Fig. 4c–i and d–i). Here, the possible interlayer-exciton-related signals generated from the heterobilayers were not found, since the post-annealing process for strong interlayer interaction was not conducted. In this study, we intended to use only individual excitons from each layer^25^. When the laser is illuminated onto the Ag-NW/MoSe_2_ region (D_1_ = 1), only MoSe_2_-excitons are coupled to the SPPs and the E_0_-related PL spectrum is collected at the Ag-NW right-end (Fig. 4c-ii and d-ii). In this process, the MoS_2_-exciton-related PL is not generated because the optical band gap of MoSe_2_ (~1.58 eV) is smaller than that of MoS_2_ (~1.88 eV). While the laser illumination onto the Ag-NW/MoS_2_ region (D_2_ = 1) only generates MoS_2_-exciton-related PL spectrum (E_1_), as shown in Fig. 4c-iii and d-iii, that onto the substrate region (D_3_ = 1) generates no optical signals (Fig. 4c-iv and 4d-iv). Figure 4e presents both E_0_ (left axis) and E_1_ (right axis) intensity plots as the output states as a function of the four different input states. The gray dashed line is the reference of the “1” state for both E_0_ and E_1_. These four combinations of output states correspond to the function of conventional a 4-to-2 binary encoder and its truth table is summarized in Figure 4f ^26^. Moreover, this architecture can also be utilized for a multi-bit multiplexer via modulating input laser positions and powers. The details are discussed in Fig. S2.

## Conclusion

In conclusion, we have proposed a concept of logic principles for plasmonic waveguides via an optical operation at room temperature. This is implemented by reconfigurable plasmon-exciton interconversion in hybrids of plasmonic nano-waveguides with TMDs arrays or stacks. The presented device architectures exhibit basic logics such as AND and XOR, as well as an advanced operation including a half-adder and a 4-to-2 binary encoder. In this work, we defined the input terminal as the laser position and the output terminal as the PL signal collection point. If we can construct the design of waveguide arbitrarily using the lithographic technique, it will be possible to clarify the input terminal distinctly likewise electronic devices. For practical applications, excitonic signals can be improved by various PL enhancement methods such as a chemical healing treatment^27^. If an electrical doping method is hybridized to this optical operation, improved signal modulation ratio^21^ and more advanced logic operations can be realized^23^. We believe that these logic principles utilizing plasmon-exciton and exciton-plasmon interconversions with simple architectures engender the development of potential applications and principles via various combinations of 2D semiconductors with versatile plasmonic waveguide architectures. Our results pave the way of 2D excitonics merged with plasmonic/photonic logic gates towards advanced nanophotonic computing.

## Methods

### Material synthesis

The monolayer TMDs were grown by our promoter-assisted method via atmospheric chemical vapor deposition (CVD)^28–30^. Prior to the growth process, the precursor solutions were prepared: ammonium heptamolybdate (Sigma, 431346) as a molybdenum precursor, ammonium metatungstate hydrate (Sigma, 463922) as a tungsten precursor, and sodium hydroxide (Sigma, 306576) as a promoter. The precursor solutions were diluted in deionized water and coated onto the SiO_2_/Si wafer by spin-casting at 3000 rpm for 1 min. The precursor-coated substrate and pure sulfur (Sigma, 344621) or selenium (Sigma, 209643) were separately introduced to a two-zone furnace. The chalcogen zone was heated up to 210 °C or 400 °C for sulfides or selenides synthesis, respectively. In the meantime, the temperature of the substrate zone was also increased to 750 ~ 800 °C with an injection of carrier gas of 500 sccm N_2_ gas. Growth conditions for each TMD monolayer were also optimized via additional gas injection of 4 sccm H_2_ and the whole growth time ranging from 17 to 25 min. The properties of the grown samples were characterized using PL and Raman spectroscopy (XperRam200VN, NANOBASE, Inc.).

### Device fabrication

The CVD-grown TMD monolayers were spin-casted with poly(methyl methacrylate) (PMMA) (950 K A4, MicroChem Corp.) and wet-transferred onto a SiO_2_ (300 nm)/Si wafer. Ag-NWs (PlasmaChem Corp.) with a diameter of ~200 nm dispersed in isopropyl alcohol (IPA) were dropped on the TMD- transferred substrates and subsequently dried for 30 s at 70 °C on a hot-plate.

### Logic operation characterization

For the optical test of devices, we used a lab-made inverted laser confocal microscope system^9,10,16^. Two different laser sources coupled to optical fibers are combined by a beam splitter and guided into the microscope. The two lasers were focused on the device with an objective lens (x100, numerical aperture, 0.9). Under the illumination of the focused lasers at the input terminals, the PL spectra were collected at the output terminals via a pinhole detector, where the laser beam and pinhole detector positions were precisely controlled with a micromanipulator. For the laser sources, three different wavelengths were used: 405 nm for the DPSS CW laser, 514 nm for the Ar-ion laser, and 633 nm for the He-Ne laser. The collected PL signals were recorded by a charge-coupled device (CCD) for imaging or guided into a monochromator equipped with a cooled CCD for spectral analysis.

## Supplementary information

Supplementary information
