# Supplementary material for: Optical logic operation via plasmon-exciton interconversion in 2D semiconductors
Source: Sci Rep. 2019 Jun 24;9:9164. doi: 10.1038/s41598-019-45204-0 (PMC6591228; doi:10.1038/s41598-019-45204-0)
Supplement: Supplementary file 1 — Supplementary information [file 41598_2019_45204_MOESM1_ESM.pdf]

Supplementary information

Optical logic operation via plasmon-exciton interconversion in 2D semiconductors

Jung Ho Kim[1,2†], Jubok Lee[1,2†], Hyun Kim[1,2], Seok Joon Yun[1,2], Jeongyong Kim[1,2], Hyun Seok Lee[3*] and Young Hee Lee [1,2*]

[1]*Center for Integrated Nanostructure Physics (CINAP), Institute for Basic Science (IBS), Sungkyunkwan University, Suwon 16419, Republic of Korea.*

[2]*Department of Energy Science, Sungkyunkwan University, Suwon 16419, Republic of Korea.*

[3]*Department of Physics, Chungbuk National University, Cheongju 28644, Republic of Korea.*

[*]Email: hsl@chungbuk.ac.kr; leeyoung@skku.edu

[†]These authors contributed equally to this work.

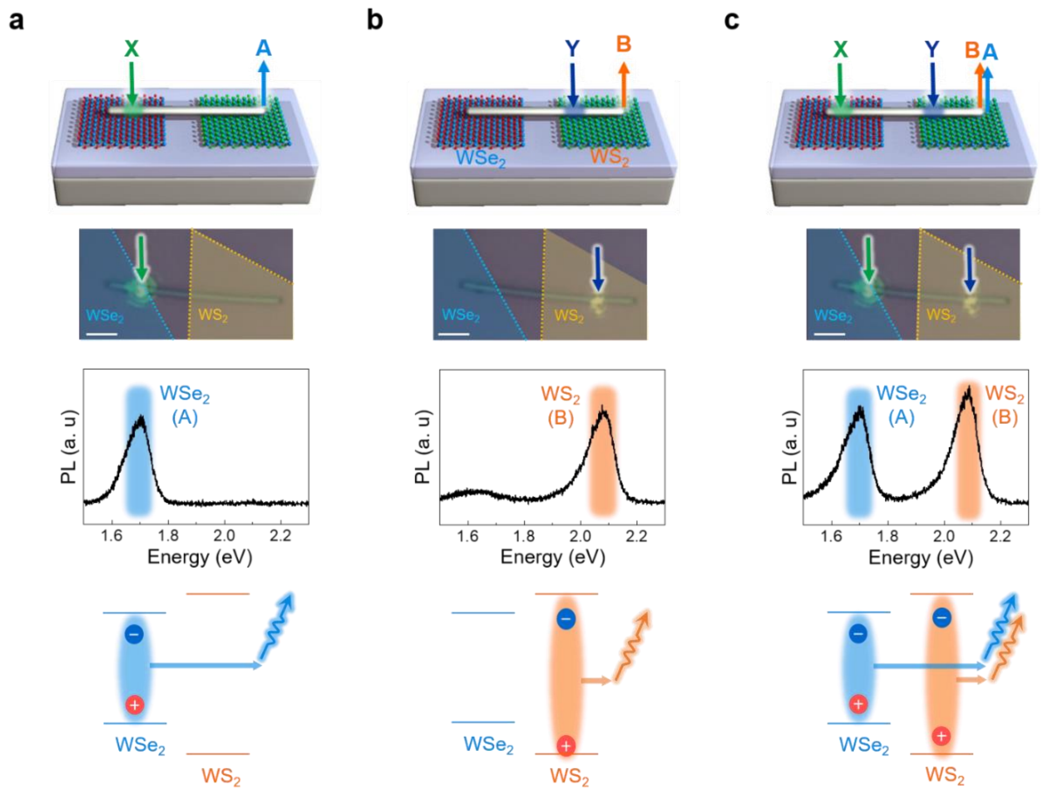

.

Figure S1. Separate PL detection mechanism in WSe_2 and WS_2 lateral heterostructure (a-c) Schematics of three combinations of laser X (green arrow, 514 nm wavelength) and Y (dark blue arrow, 405 nm wavelength) illumination on Ag-NW bridged WSe_2-WS_2 device (top panel). The related OM image and PL spectrum for each combination (middle panel). WSe_2-exciton-coupled SPP passes WS_2 without absorption since the optical band gap of WSe_2 (~1.7 eV) is smaller than that of WS_2 (~2.08 eV). Therefore, when each material is excited, the corresponding PL signal is separately and simultaneously detected (bottom panel). Scale bars in the optical images indicate 5 μm.

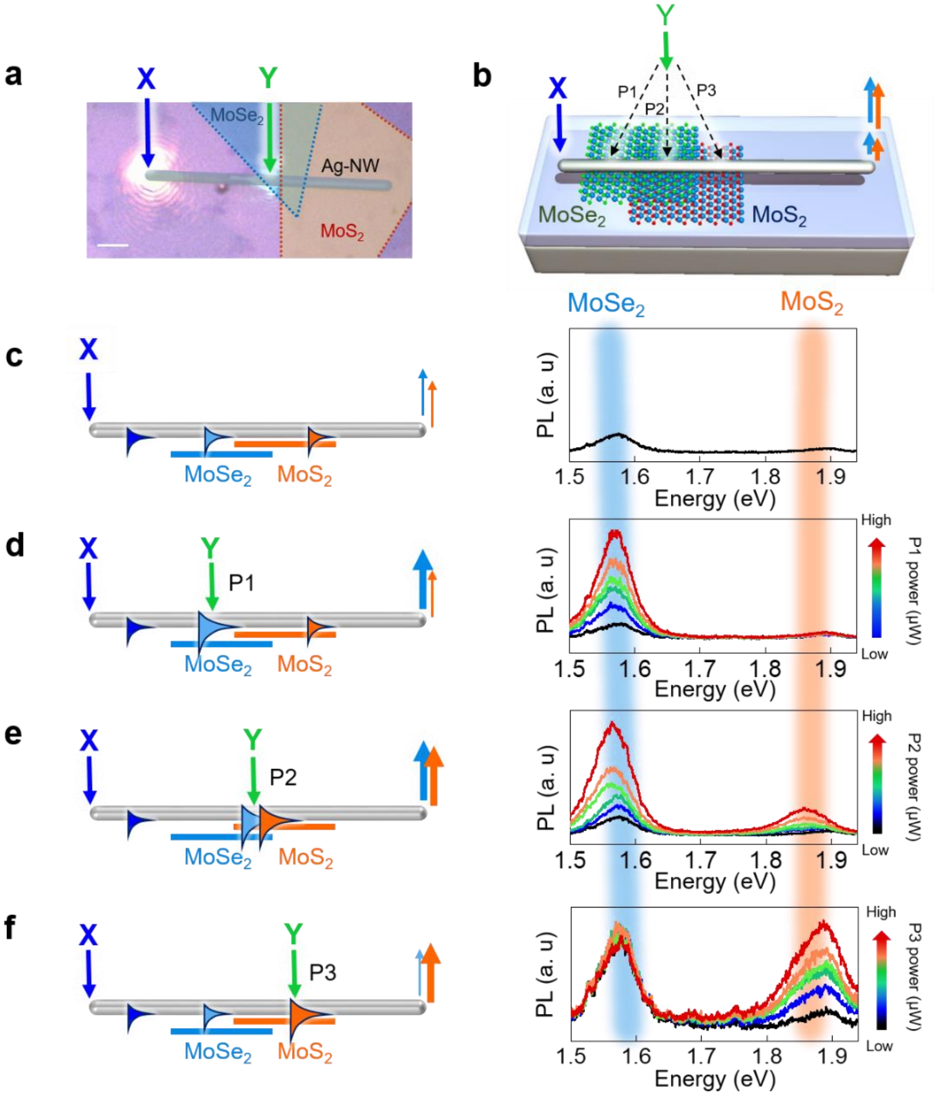

Figure S2. Multi-bit multiplexing. (a,b) OM image and schematic illustration of Ag-NW on heterostacked $MoSe_2/MoS_2$. Multiple excitons are modulated by laser illumination positions. The scale bar in the optical images indicates 5 μm. (c) When only laser X (dark blue arrow, 633 nm wavelength) is applied, this excites both materials and shows a small intensity. (d-f) When laser Y (green arrow, 514 nm wavelength) is positioned at different positions (P1=$MoSe_2$, P2=heterojunction, and P3=MoS_2, respectively) corresponding PL peak is selectively excited. Furthermore, by varying control laser position and power, we can selectively modulate PL intensity.
